# Editorial

**Published:** 1839-11-02

**Authors:** 


### THE MEDICAL EXAMINER.

### PHILADELPHIA, NOVEMBER 2, 1839.

In the transactions of the Pathological Society will be found a highly interesting report, by Dr. Pennock, upon the action and sounds of the heart. This is the best evidence that the objects of the society are very far from being limited to the cultivation of pathological anatomy, a most useful aid to the science of medicine, but still a mere assistant, and not the leader in the study.

The experiments of Dr. Pennock were conducted upon a scale of expense, and prosecuted with a degree of care, which require a pure devotion to science, possessed by but few individuals. It is not our practice nor our present purpose to write editorials in praise of the exertions of particular individuals, more especially when they are to be found in our own city, and amongst our own friends; but when a series of expensive and troublesome experiments has been completed, we are not only gratified, but, as it were, are obliged to add our testimony to the fidelity and disinterested care of the observers. Every precaution, we know from our own observation, was taken to avoid error; and when the result of an experiment seemed at all doubtful, it was repeated again and again until the apparent obscurity was fully removed.

It will be perceived by a reference to the experiments, that besides confirming several points already set forth by the British experimenters, the experiments of Dr. Pennock have developed some new facts. These are the existence of an auricular sound, faint indeed, and to a great degree merged in that of the ventricles, but still real. Indeed, when the fact of real muscular contraction of the auricles is proven, we know from analogical reasoning that sound, which is the necessary consequence of muscular contraction, must be produced. The active muscular contraction of the auricles has been doubted by some physiologists, we hardly know why, for the evident muscular fibres of the auricles are certainly designed for no other purpose than other muscles, that is, contraction. The experiments of Drs.

Pennock and Moore have set this question also at rest, and proved satisfactorily to all present, that the muscular fibres of the auricles were in action during their systole.

There are two other points ascertained positively by these observers, from direct experiment, which we believe were not noted in previous observations of the kind. These are—1st, that the ventricular sound on the right is much sharper and clearer than that of the left, which is more dull and prolonged; 2d, that the second or valvular sound ceases by the congestion of the ventricles, ceasing first on the right side. With both of these facts we have been long familiar, from our observations of the sounds of the heart in the living body, both in the healthy and diseased condition of this organ; and it is not a little gratifying to us to find that the clinical observations which we have often made and pointed out to our pupils have been abundantly confirmed by the sure test of direct experiment. This observation throws light upon a large number of pathological facts, which are often with difficulty understood without knowing the effect of congestion upon the heart.

The last point of interest which has been for the first time explicitly stated, is the less degree of loudness of the second sound over the pulmonary artery and valves than over the aorta. This probably arises from the pulmonary valves being so much weaker and thinner.

---

The system of medical education in this country has, of late years, rapidly been losing the character of centralization. Philadelphia is no longer the common alma mater of American physicians. Her schools still flourish, and the absolute number of students who resort hither has rather increased than diminished; but other good schools have risen up in different sections, and Philadelphia must now be content if she can make good her claim to be considered *prima inter pares.* Doubts have been entertained by some as to the policy of the expanding system; but the current of opinion runs strongly the other way, and little difficulty is now experienced in obtaining charters for as many faculties as ask for them. The advantages of this liberal policy are numerous and solid; there are some evils incident to it, many of which time will correct, while a few are inherent in the system. It cannot be denied that the talent scattered through two or three faculties might be more effective if concentrated in a single body; and it is plausibly urged, that ephemeral schools are got up, without any sure prospect of permanency and stability. The first of these objections has much apparent, some real weight. A union of all the talent in one body would be an excellent thing, but it is by no means certain that, if a single institution monopolized the field, talent would be the only passport of admission to vacancies. Again, if faculties prove short-lived, they chiefly hurt themselves, while the *prestige* attached to older institutions is a strong check upon the needless multiplication of new ones. The benefits of the free system are, we think, manifold. By introducing competition, it improves the plan of instruction, and stimulates the lecturer to constant exertion, while it removes a source of ill-feeling by opening to all a participation in the honourable and lucrative employment of teaching.

It has been proposed to bring about these good results by a comprehensive act of incorporation, superseding the necessity of repeated legislative interference in the creation of charters. Such is the plan of the Medical College Bill, which we publish to-day. The objects of this bill are excellent. How far the working of the details will succeed in carrying out these objects, time only can show. The appointment of a Board of Examiners, enjoying the entire confidence of the profession, and the establishment of a fee for graduation not exceeding that required by the organized colleges, seem to be proper preliminary steps, which we doubt not will be adopted.

The Medical Faculties, already in existence in this city, will, we presume, for some time at least, continue to occupy their present footing. It is not probable that students who attend their lectures, will resort elsewhere for degrees; and we think it likely also that any new combination of teachers, that may wish to start, will endeavour to obtain a charter to confer degrees. They will be unwilling to surrender the graduating fee, and will naturally be anxious to enjoy the privileges of the institutions with which they compete. Hence we doubt whether the Medical College can go into extensive operation, so long as the other faculties retain the power to grant degrees.

